# Editorial: Current progress and challenges in psychopharmacology research

**DOI:** 10.3389/fphar.2024.1504943

**Published:** 2024-10-31

**Authors:** Marna Eliana Sakalem, Tharcila V. Chaves, Vinicius Antônio Hiroaki-Sato, Miriane de Oliveira, Ricardo Tabach

**Affiliations:** ^1^ Department of Anatomy, State University of Londrina (UEL), Londrina, Brazil; ^2^ Medical School, Pontifical Catholic University of Parana, Londrina Campus, Londrina, Paraná, Brazil; ^3^ Amsterdam University Medical Centers, Amsterdam, Netherlands; ^4^ Onkos Molecular Diagnostics, Ribeirão Preto, Brazil; ^5^ Federal University of São Paulo, São Paulo, São Paulo, Brazil; ^6^ Universidade Santo Amaro, São Paulo, São Paulo, Brazil

**Keywords:** psychobiology, hallucinogens, psycotropic drugs-farmacokinetics, farmacology, mental disorder

Several reasons could be raised to demonstrate the maintenance of psychopharmacology as one of the most intriguing and challenging areas of research. Mental health and agents with central effects are a crescent topic of interest. Innovative research methods will be responsible for a further boost in the understanding of neurotransmission and mechanisms of action of psychotropic agents, leading to the discovery of new treatments that could aid the suffering mind. Even if we consider how far scientists have brought psychopharmacology up to this point, the field is still blooming, and there is a long road ahead of us.

Human beings have always been fascinated by compounds that could affect their minds, either with the aim of putting the psyche in a comfortable zone or altering consciousness. It was in the beginning of the last century that the word psychopharmacology (derived from the Greek *psykhē*, i.e., mind, spirit or soul, and *farmakon*, i.e., poison or drug, and *logos*, i.e., study or knowledge) was first used. The breakthrough of modern psychopharmacology started in the 1950s, with the synthesis of chlorpromazine, an antipsychotic drug, and continued with the discovery of all psychotropic drug classes. This big start was only possible due to researchers investigating the mechanisms of action of diverse drugs, including several that were at first used for other purposes, but happened to also influence the nervous system. The following decades presented a constant and rapid expansion, which enabled scientists not only to pharmacologically interfere with a dysfunctional brain, but also to use psychotropic drugs to understand how the brain works. More importantly, multiple disorders, such as mood and psychiatric disorders, were finally acknowledged and could be more precisely treated.

The World Health Organization (WHO) assumes that untreated mental disorders account for 13% of the total global burden of diseases, and it has been estimated that, by 2030, depression will be one of the leading causes of disability worldwide, surpassing heart disease and cancer. Recently, the pandemic situation led to increased anxiety and fear of uncertainties, all of which have a negative effect on maintaining mental health.

Psychiatric conditions and chronic pain can be difficult to manage with conventional pharmacological treatments and it is not uncommon to have patients who do not respond to several pharmacological options. Therefore, there is a need (and even an urgency) for developing new approaches and treatments to medical conditions that affect the central nervous system.

The articles selected for this Research Topic bring compelling information on this matter. Two of them are focused on psychedelic science, a field that has been growing intensively in the past decades, with the publication of many auspicious results when these drugs are administered in a controlled and safe setting. For instance, the popular anaesthetic ketamine is also being used as an antidepressant in cases of treatment-resistant depression. The bibliometric analysis performed by Wang et al. offers valuable insights into trends, key contributors and thematic focus areas within the ketamine research field. Moreover, Dornbierer et al. have investigated new routes of administration for the South American psychedelic brew called ayahuasca, which can present beneficial effects on people struggling with affective disorders. In an open-label within-subject trial in 10 healthy male participants, Dornbierer et al. have observed fewer side effects with peroral ayahuasca-analogue formulations.

Still on the plant-as-medicine issue, the studies performed by Nguyen et al. and Hasan et al. are providing some food for thought. Nguyen et al. have detected antidepressant and anxiolytic effects produced by the essential oil from *Citrus reticulata* Blanco. With an intricated study design, the authors state that these effects may be due to the potential of some compounds to act against neuroinflammation and neurodegeneration. On a neuroprotection approach, Hasan et al. performed a review on phytochemicals (from fruits, vegetables, spices, teas and herbs) in the management of traumatic brain injury. They conclude that although the use of phytochemicals in the context of traumatic brain injury seems promising, efficacy and safety studies on human beings are still lacking.

The Perspective article by Furqan discusses the recent approval by the US Food and Drug Administration (FDA) of the first drug for the treatment of Rett syndrome. This is a rare sporadic neurodevelopmental disorder characterised by normal early growth and development, followed by regression in previously acquired activities, primarily affecting motor, cognitive and communication skills. From 2 years of age, children who live with this syndrome are now eligible to be treated with trofinetide.

Lastly, focusing on efficacy and safety of fospropofol disodium sedation, Zhao et al. describe their study protocol. The authors aim to randomise 256 participants who are scheduled for same-day bidirectional endoscopy under sedation in 2 groups: propofol group (control) and fospropofol group. Their hypothesis is that the efficacy and safety of fospropofol sedation will not be inferior to that of propofol.

With this constellation of psychopharmacological topics, we expect to provide the readers with assorted information on this field. The progress is evident; the challenges reside in making this progress safe and accessible to everyone who may need it.

